# Editorial: Metabolomics in crop research – current and emerging methodologies, volume II

**DOI:** 10.3389/fpls.2023.1292878

**Published:** 2023-09-22

**Authors:** Marta Sousa Silva, Ute Roessner, Carlos Cordeiro

**Affiliations:** ^1^ Laboratório de FTICR e Espectrometria de Massa Estrutural, Faculdade de Ciências, Universidade de Lisboa, Lisbon, Portugal; ^2^ Biosystems and Integrative Sciences Institute, Faculdade de Ciências, Universidade de Lisboa, Lisbon, Portugal; ^3^ School of BioSciences, The University of Melbourne, Melbourne, VIC, Australia

**Keywords:** metabolomics, agriculture, biomarkers, volatile organic compounds, pathogen resistance, species discrimination, new products discovery

Metabolomics’ potential in plant and crop research is immense, thus giving an important contribution to improve crop yield and quality, to understand plant development and response to the environment, to pathogens, to assess food safety and maximize agriculture profits. This is the second edition of the Research Topic “*Metabolomics in Crop Research - Current and Emerging Methodologies*”. It contains 10 articles from 81 authors that highlight the current methodologies and applications of metabolomics for the discrimination of crop species and cultivars, in the analysis of a crop’s response to biotic and abiotic stresses, in the characterization of important compounds as well as the discovery of new compounds and metabolic pathways.

Crop domestication results from the selection and management of plant phenotypes over time, to achieve the desired characteristics. The domestication of *Capsicum annuum* (chili pepper), which started 6,000 years ago, caused considerable changes in the fruit and consequent alterations in metabolism. Cervantes-Hernández et al. analyzed the metabolic profiles during fruit development of wild and domesticated accessions of *C. annuum* L. Both groups were clearly separated based on their metabolic profiles and significant differences were observed in the fruit development between the wild and domesticated plants. This work highlights the effect of the domestication process in plant metabolism, contributing in the future to the development of plant breeding strategies to restore metabolic diversity. *Vitis vinifera* (grapevine) is another successful case of plant domestication, with the development of nearly 6,000 different varieties, grouped according to their geographic-genetic origin (GEN-GEO groups). Šikuten et al. were able to discriminate grapevine varieties belonging to different GEN-GEO groups based on their grapes’ volatile metabolic profiles. This analysis allowed not only genetic discrimination, but also the identification of the country of origin, thus contributing to the promotion of grape quality and value linked to geographical origin. One of the main problems in grapevine cultivation is plant susceptibility to several diseases, particularly downy mildew. In the work of Ciubotaru et al., the metabolic responses to the inoculation with *Plasmopara viticola* (downy mildew infectious agent) in different resistant genotypes were analyzed, following a post-infection time-course analysis. Several potential resistance biomarkers were identified, thus contributing to understanding grapevine resistance mechanisms and assisting future breeding programs for improving its resilience to infection.

The use of plants in traditional Chinese medicine is a millenary practice that is becoming widespread. Species discrimination is crucial since they can have different pharmacological effects. This is the case of *Polygonatum*, whose rhizomes are widely used in traditional Chinese medicine. Three *Polygonatum* species are included in the *Chinese Pharmacopoeia* (2020 Ed.), each one with different medicinal effects. Since their discrimination is often based on physiology and morphology, Wang et al. analysed the metabolic profile of these *Polygonatum* species and identified the discriminant compounds between them, thus contributing for a more accurate species identification and paving the way for a better understanding of its mechanism of action.

*Oryza sativa* (rice) is one of the most important food crops in the world. Hence, it is important to provide the best agricultural conditions for its growth. The use of volatile organic compounds produced by specific bacteria is a promising strategy to increase productivity in a more sustainable way. Almeida et al. analysed the metabolic composition and effects of bacterial volatilome on the growth and metabolism of rice and identified two promising bacterial isolates that improve rice growth, contributing to a more sustainable agriculture. Given the importance of rice, experiments have been carried out to understand the effects of deep space flight on rice seed transport and cultivation. Having in mind that spaceflight stress affected rice growth, leading to significant changes in metabolic pathways, Zeng et al. analysed the metabolome and proteome of F2 generation plants grown from seeds exposed to this stress. Rice F2 generation plants preserved the memory of spaceflight stress, thus bringing a new perspective for these studies in other crops and its potential for space flight and human colonization beyond Earth. A popular substitute to rice is quinoa (*Chenopodium quinoa* Willd.) given its high nutritional value. Although still considered a minor crop, the Food and Agriculture Organization (FAO) gave it the statute of a promising crop for the human diet that can contribute to food security in the 21^st^ century. In the defense response against microbes, herbivores, and insects, quinoa plants produce the triterpenoid saponin. Zhao et al. studied this compound’s biosynthesis pathway in quinoa using a combined transcriptomics-metabolomics strategy. These discoveries contributed to a thorough characterization of the metabolic mechanism of triterpenoid saponin in quinoa. Besides biotic stresses, quinoa is affected by drought stress. Huan et al. studied the drought tolerance response mechanisms in quinoa after drought and rewatering treatments, using a transcriptomics and targeted metabolomics approach. This work will impact future breeding programs of new drought-tolerance quinoa varieties.

Leaves from coffee (*Coffea* genus) plants, and others from the same family (*Rubiaceae*), produce highly important bioactive polyphenols, with applications in food and pharmaceutical industries. Leaves are a disregarded by-product of the coffee industry, which can be used for the extraction of these relevant compounds. Castro-Moretti et al. developed a single-step extraction methodology for the recovery of a vast diversity of phytochemicals from coffee and other *Rubiaceae* leaves and a method for quantification and detection of new polyphenols. This research will impact future breeding programs, boost phytochemical discovery and contribute to the circular economy, reducing waste and increasing value. The discovery of new compounds from the hemp seed (*Cannabis sativa* L.) is also quite relevant, given its applications in the pharmaceutical, nutraceutical and food industries. Padilla-González et al. analyzed the metabolic profile of several hemp seed accessions to identify new metabolites and associate specific biologically important molecules to particular plant accessions. Once again, these results can be used in future optimization of breeding programs for the development of new and improved varieties.

Metabolomics application to plant and crop research is constantly progressing. The resulting information can be used in the development of new improved crops, able to respond to climate change, environmental degradation, pathogen attacks, and loss of biodiversity. Metabolomics must be part of the agricultural research and innovation strategy towards a sustainable production. This is the Volume II of the Research Topic “*Metabolomics in Crop Research - Current and Emerging Methodologies*”, and we are looking forward to future contributions in the next edition.

## Author contributions

MS: Conceptualization, Writing – original draft, Writing – review & editing. UR: Writing – review & editing. CC: Writing – original draft, Writing – review & editing.

